# Bacterial carrier-mediated drug delivery systems: a promising strategy in cancer therapy

**DOI:** 10.3389/fbioe.2024.1526612

**Published:** 2025-01-08

**Authors:** Sizuo Yan, Yu Gan, Huizhe Xu, Haozhe Piao

**Affiliations:** ^1^ Department of Neurosurgery, Liaoning Cancer Hospital and Institute, Cancer Hospital of China Medical University, Cancer Hospital of Dalian University of Technology, Shenyang, China; ^2^ Institute of Cancer Medicine, Dalian University of Technology, Dalian, China; ^3^ Central Laboratory, Liaoning Cancer Hospital and Institute, Cancer Hospital of China Medical University, Cancer Hospital of Dalian University of Technology, Shenyang, China

**Keywords:** bacteria, bacterial derivatives, drug delivery, bacteriotherapy, cancer

## Abstract

Cancer is a major killer threatening modern human health and a leading cause of death worldwide. Due to the heterogeneity and complexity of cancer, traditional treatments have limited effectiveness. To address this problem, an increasing number of researchers and medical professionals are working to develop new ways to treat cancer. Bacteria have chemotaxis that can target and colonize tumor tissue, as well as activate anti-tumor immune responses, which makes them ideal for biomedical applications. With the rapid development of nanomedicine and synthetic biology technologies, bacteria are extensively used as carriers for drug delivery to treat tumors, which holds the promise of overcoming the limitations of conventional cancer treatment regimens. This paper summarizes examples of anti-cancer drugs delivered by bacterial carriers, and their strengths and weaknesses. Further, we emphasize the promise of bacterial carrier delivery systems in clinical translation.

## Graphical Abstract

**Figure:**
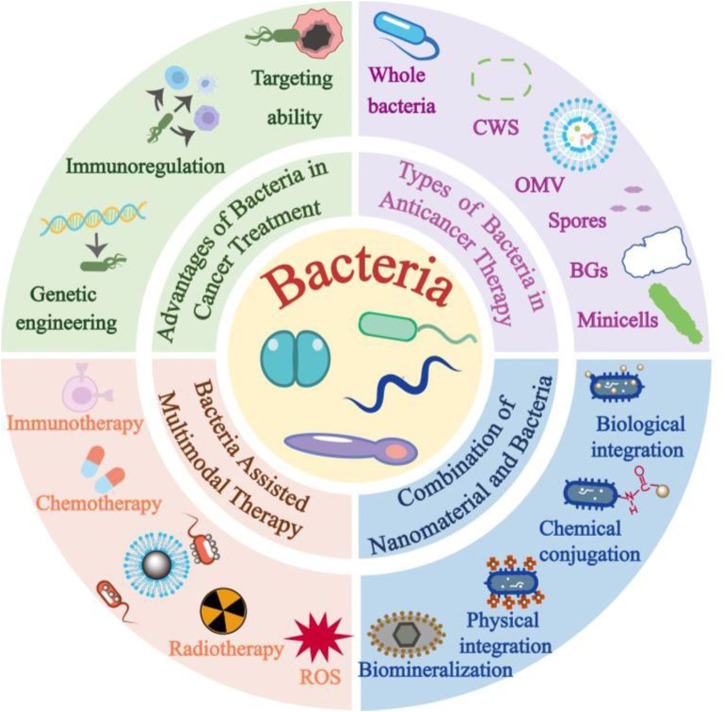
Schematic illustration of the application of bacterial carriers in anti-tumor therapy, including the advantages of bacterial carriers in cancer treatment, the classification of bacterial carriers, bacteria combined with nanomaterials for cancer therapy, and the strategies for constructive integration of bacteria-nanomaterials.

## 1 Introduction

Despite the tremendous advances in medical technology, cancer remains an insurmountable challenge. According to the WHO, in 2022, there were nearly 20 million new cases of cancer and 9.7 million deaths from cancer worldwide (Ferlay et al., 2024; Bray et al., 2024). It is estimated that about one-fifth of people will develop cancer in their lifetime, while about one in nine men and one in twelve women will die of cancer. Demographically based projections indicate that by 2050, there will be 35 million new cases of cancer (Bray et al., 2024). Furthermore, cancer is not only a serious threat to people’s health but also carries significant social and macro costs (Chen et al., 2023). At present, conventional treatments for cancer include surgery, chemotherapy, and radiotherapy. In addition, there are new therapies such as gene therapy, immunotherapy, and stem cell therapy (Mathis et al., 2019). Despite the good progress made in all these cancer treatments, each has its limitations and side effects (Maman and Witz, 2018), and therefore it has become necessary to develop a more promising solution.

Drug Delivery Systems (DDS) are innovative and viable therapeutic modalities that fully regulate the dissemination of drugs in the body concerning dosage, space, and time (Park et al., 2022). The use of nanomaterials as drug carriers to release drugs into target tissues or cells through specific pathways is a new type of drug delivery technology. This technology can increase drug utilization, improve therapeutic effects, reduce costs, and minimize side effects, and has now become the forefront of research in medicine. Currently, common drug nanocarriers can be broadly categorized into lipid nanoparticles, polymer nanoparticles, and inorganic nanoparticles, with lipid nanoparticles and polymer nanoparticles being the most commonly used drug delivery carriers. However, these delivery systems suffer from poor stability and barrier penetration, low cellular uptake, and difficulty assembling functional components, making it difficult for therapeutic agents to reach the tumor site (Cao and Liu, 2020). Therefore, developing new drug delivery carriers to improve the effectiveness of antitumor therapy has become crucial.

**FIGURE 1:**
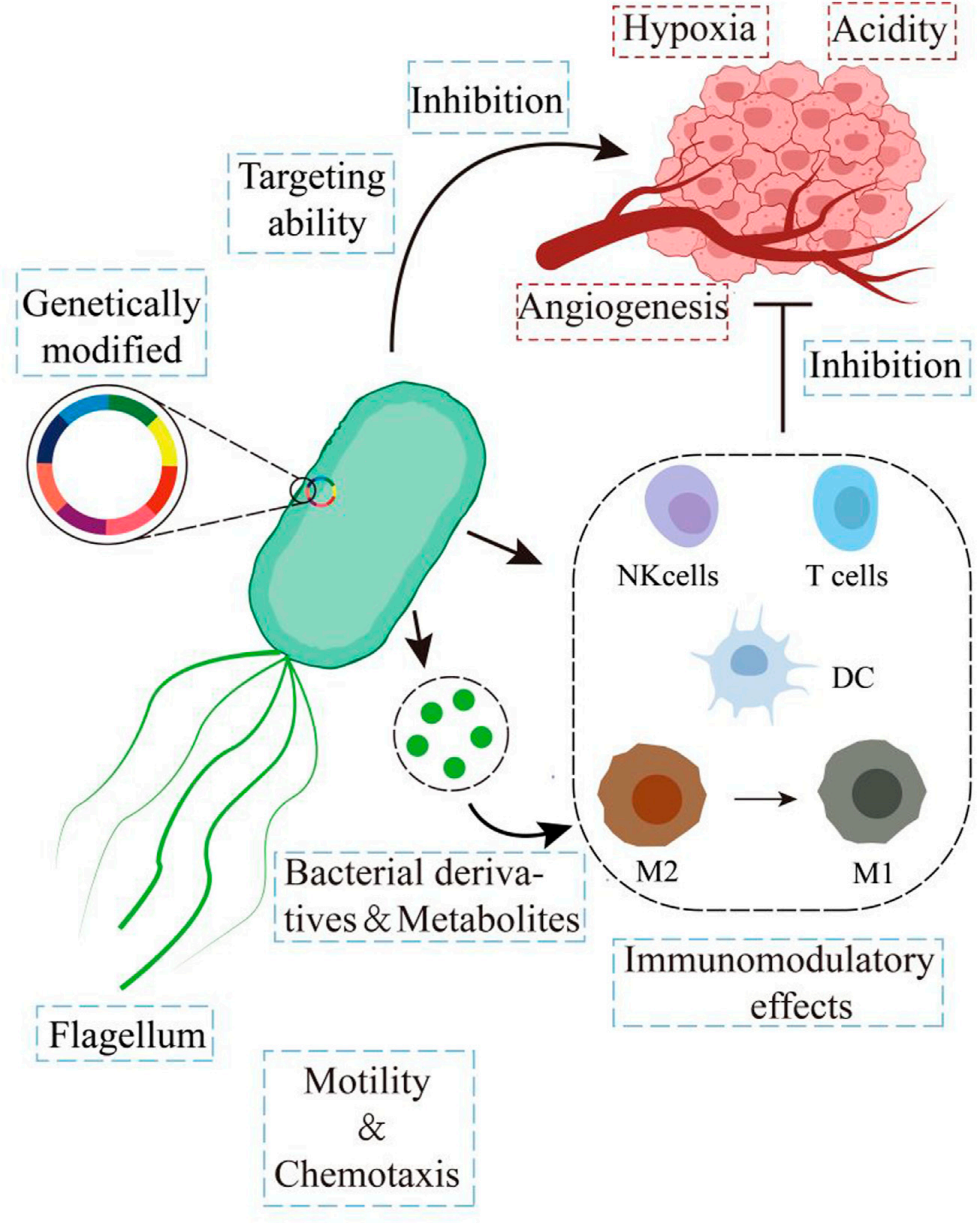
Diagram illustrating the advantages of bacteria in anti-tumor therapy.

Bacteria are the oldest, most abundant, and most widely distributed organisms in the world, colonizing various physiological organs in the human body and regulating organ function (Cubillos-Ruiz et al., 2021; Tamburini et al., 2016; Stewart et al., 2018). As early as the 18th century (Starnes, 1993; Cann et al., 2002), it was recorded that certain bacterial infections in cancer patients with malignant disease could be relieved. In the early 19th century, Vautier recorded that tumors receded in patients who suffered from gangrene (Mowday et al., 2016). Subsequently, during the late 1800s, William Coley used *Streptococcus pyogenes* infections to regress patients’ sarcomas, which inaugurated bacterial-mediated microbial therapy (Coley, 1893). The results showed that various species of bacteria such as *Streptococcus*, *Salmonella*, *Escherichia coli*, *Clostridium*, *Bifidobacterium, Listeria*, and *Lactococcus* were effective in anticancer therapy (Yin et al., 2022). Some bacterial-based treatment regimens are also widely used for their excellent efficacy: for example, *Mycobacterium* bovis *bacillus* Calmette-Guérin (BCG) vaccine, which has been successfully used in the therapy of non-muscle invasive bladder cancer (Pettenati and Ingersoll, 2018). In the mid-1990s, recombinant DNA technology produced more efficient, safer, and functionally abundant engineered bacteria (Anderson et al., 2006). However, their application has been limited due to lag, uncontrollability, and inadequate targeting efficiency. Nowadays, with the rise and advancement of nanomaterials science and synthetic biology technology, the safety and efficacy of bacterial therapies have continued to improve, rekindling the enthusiasm for bacterial therapies (Lin et al., 2021; Redenti et al., 2024; Scott et al., 2017; Solomon et al., 2015). DDS based on bacterial carriers is an innovative and promising approach. Bacterial carriers include bacteria and bacterial derivatives. Compared to synthetic materials such as liposomes and polymers, bacteria, and their derivatives have many attractive properties. Firstly, bacteria have good tumor colonization ability and can accumulate at the lesion site. Moreover, bacteria are biocompatible and can pass through the body’s physiological barriers. In addition, bacteria have the advantage of being easy to manipulate for gene editing and their derivatives can cause immune activation. The flagellum of bacteria makes them chemotaxis, allowing for more efficient drug delivery and a quantum leap in anticancer therapeutic efficacy (Figure 1) (Wu et al., 2022).

In this article, we summarize the advantages of bacterial treatment of tumors and show the classification of bacterial carriers and examples of the delivery of anticancer drugs by various types of carriers. In addition, we concentrate on the application of combining nanotechnology with bacterial carriers in antitumor therapy. This work also summarizes current strategies for combining nanoparticles with bacteria and bacterial derivatives. Finally, we emphasize the advantages and disadvantages of bacterial carriers when used to treat cancer and their potential for clinical translation.

## 2 Advantages of bacteria in cancer treatment

### 2.1 Targeting the tumor microenvironment (TME)

The TME includes hypoxia, acidic pH, angiogenesis, and immunosuppression. Due to the rapid growth of tumor tissues with active metabolism, it increases its oxygen demand. At the same time, the vascular system in and around the tumor is disorganized and irregular, resulting in inadequate oxygen diffusion (Jain, 2013). Therefore, the central area of the tumor presents a necrotic, hypoxic environment with an oxygen partial pressure as low as 7–28 mmHg, whereas the oxygen partial pressure of normal tissue is 40–60 mmHg (West and Slevin, 2019). Bacteria, as natural prokaryotic cells, can actively migrate into the hypoxic TME by their autonomous propulsion and anaerobic properties (Kasinskas and Forbes, 2006). Live facultative/obligate anaerobes such as *E. coli, Salmonella,Bifidobacterium bifidum,* and *S. pyogenes* can sense hypoxic environments by using their chemoreceptors, which are naturally targeted to hypoxic and necrotic areas of tumors (Kucerova and Cervinkova, 2016).

Due to insufficient oxygen supply, tumor cells are limited to metabolizing energy through anaerobic fermentation, which leads to lactic acid accumulation and the formation of an acidic tumor microenvironment (Kumari et al., 2021). Tumor acidic environment can also induce bacterial-specific gene activation. Flentie et al. (2012) co-cultured *Salmonella* with melanoma cells or colon cancer cells and identified 5 genes that can be activated specifically by cancer cells. Further studies revealed that the activation of these genes may be caused by the acidic microenvironment of the tumor cells, suggesting that the bacteria are capable of responding positively to the acidic TME. Tumor cells release a substantial quantity of vascular endothelial growth factor during growth, inducing the formation of chaotic and irregular tumor-specific blood vessels. These blood vessels can enclose the bacteria, not only promoting bacterial colonization but also providing an adequate supply of nutrients to the bacteria, which is conducive to bacterial proliferation (Khalaf et al., 2021). Hypoxia also leads to suppression of local immune cell function resulting in an immunosuppressive microenvironment (Chouaib et al., 2017). In tumor tissues, the ability of immune cells to recognize and kill bacteria is inhibited, which provides a favorable environment for bacterial survival. It has been shown that bacteria are more likely to survive in a tumor-immunosuppressive environment (Stritzker et al., 2010; Tian et al., 2022).

In conclusion, the characteristics of the TME and the interactions between bacteria and tumors are not only important for the natural targeting of bacteria to tumors but also provide a basis for modifying bacteria and improving their targeting ability.

### 2.2 Colonization ability

Most bacteria used for tumor therapy have flagella, which mainly perform chemotaxis, motile, and invasive functions. These functions performed by the flagellum can help the bacteria to better penetrate the intratumor tissue, which is crucial for bacterial colonization in tumors (Min et al., 2008; Zhao et al., 2005; St Jean et al., 2008; Kasinskas and Forbes, 2007).

### 2.3 Immunomodulatory effects

Bacteria and their secreted metabolites such as peptidoglycan, and lipopolysaccharide (LPS) augment the antigenicity of tumors and provide potent immunostimulatory signals. They combine with pattern recognition receptors (PRRs) expressed by immune cells such as dendritic cells (DCs) and macrophages, initiating an immune response that allows the immune system to recognize and kill tumor cells. Bacteria and their metabolites also initiate an adaptive immune response to kill tumor cells via natural killer cells, CD4^+^, and CD8^+^ T cells (Zitvogel et al., 2016).

Furthermore, certain components in bacteria can also cause phenotypic transformation of immune cells. Flagellin was found to convert pro-tumor macrophages into anti-tumor macrophages and to transform the immunosuppressive microenvironment into an environment of normal immune function (Zheng et al., 2017).

### 2.4 Can be genetically engineered to enhance therapeutic functions

Genetic engineering of bacteria to develop safer and more effective cancer treatments is a major focus of bacterial anticancer therapies. Bacterial genetic modification can reduce bacterial cytotoxic effects to improve safety (Hill et al., 2011)and enhance bacterial targeting, invasiveness, and tumor cell killing (Yoon et al., 2017).

In addition, synthetic biology methods to implant sensing and responsive genetic circuits in bacteria enable bacteria to synthesize or release drugs in response to specific sensing stimuli. Moreover, combining multiple response circuits with bacteria through synthetic biology approaches could enable engineered bacteria to respond to the complex physiological environment of the organism. This allows for better targeting and control of the bacterial carrier delivery system, enabling precise drug delivery and reducing drug side effects (Feng et al., 2023).

## 3 Types of bacteria in anticancer therapy

### 3.1 Whole bacteria

Whole bacteria can be categorized into inactivated and live bacteria, which can be improved through genetic engineering or other technological methods to enhance their ability to deliver drugs to treat cancer.

**FIGURE 2:**
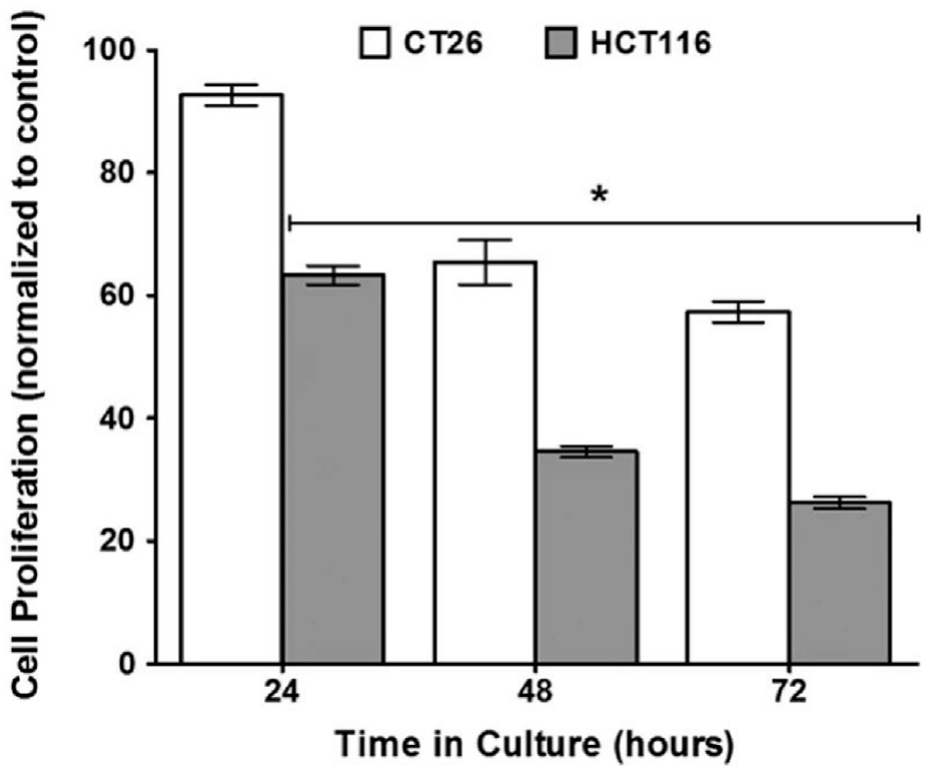
Diagram illustrating that inactivation of *Clostridium sporogene*, a naturally tumorigenic bacterium, significantly reduced the proliferation of CT26 and HCT116. Adapted from reference (Bhave et al., 2015). Nature, Copyright 2016.

Inactivation of bacteria is a special inactivation technique that prevents the bacteria from growing and reproducing while retaining their original structure and characteristics. Records of inactivated bacteria for the treatment of cancer date as far back as the advent of “Coley’s toxin” in the 19th century, when William Coley injected inactivated *S. pyogenes* and *Serratia marcescens* into sarcoma patients (Hoption Cann et al., 2003). Following Coley’s work, Teoh’s laboratory demonstrated that the inactivation of *Clostridium sporogene*, a naturally tumorigenic bacterium, significantly reduced the proliferation of CT26 and HCT116 colorectal cancer cells to 57.3% and 26.2%, respectively (Figure 2) (Bhave et al., 2015). However, inactivated bacteria often suffer from insufficient anti-tumor effects. To address this problem, Yang and Xuan incorporated 125I/131I into inactivated bacterial carriers, which can prolong the presence of radioactive iodine at the site of the lesion, thus enabling internal radioisotope therapy (IRT) of the tumor (Pei et al., 2022).

In contrast to inactivated bacteria, live bacteria can actively target most tumors, including metastases (Forbes, 2010), and most live bacteria can better penetrate and colonize intratumoral tissues depending on the flagellum (Felgner et al., 2018). However, the toxicity and uncontrolled immunogenicity of the bacteria greatly limit the use of live bacterial therapies in practice. Besides, bacteria can suppress macrophage migration and disrupt the integration of the barrier established by epithelial cells, thereby allowing tumors to diffuse and invade surrounding healthy tissues (Gagnaire et al., 2017). Therefore, improving the safety of bacterial therapy has become a key issue in bacterial cancer therapy.

The attenuated modification of bacteria is a common method to improve the safety of bacterial therapies. The BCG vaccine is a live bacterial preparation made from attenuated suspensions of *Mycobacterium bovis* by Charles Calmette and Camille Guérin in 1900 (Kamat et al., 2015). When they cultured *M. bovis* in bile-containing media, they found that the bacterial virulence gradually decreased and after more than 1,000 passages the attenuated strain that we use today was obtained. Bladder instillation of BCG is a standard treatment option for patients with high-risk non-muscle invasive bladder cancer in the clinic. Compared with traditional means of inactivation and attenuation, obtaining attenuated bacteria by knocking out virulence factors through genetic engineering techniques not only reduces the pathogenicity of the bacteria but also preserves their tumor-targeting properties. For example, an attenuated *Salmonella typhimurium* strain (named VNP20009) obtained by deletion of the purI and msbB genes not only dramatically improved safety, but also promoted their accumulation in the tumor tissue (Toso et al., 2002).

Subsequently, a large number of researchers have concentrated on attenuating the virulence of various bacteria, including *Salmonella*, *Clostridium*, *E. coli*, and *Listeria*, through genetic and chemical modifications (Felgner et al., 2018; Mercado-Lubo et al., 2016; Fritz et al., 2016; Dang et al., 2001; Chowdhury et al., 2019; Zhou et al., 2018). In addition, engineered bacteria are a promising carrier for drug delivery, and today substantial studies are using engineered bacteria for targeted delivery of interfering RNA (Jiang et al., 2007), DNA vaccines (Kong et al., 2012), proteins (Binder et al., 2013), and small molecule therapeutics (Xie et al., 2017) for the treatment of malignant tumors. Protein therapeutic molecules, including antibodies (Daassi et al., 2020), inflammatory factors (Schmitt and Greten, 2021), metabolism-blocking proteins (Liu et al., 2017), enzymes (Yu et al., 2024), and apoptosis-inducing agents (Hu and Kavanagh, 2003) have also been successfully combined with engineered bacteria for targeted therapy of tumors (Table 1).

**TABLE 1:** Application of bacterial delivery of anticancer drugs in cancer treatment.

Bacteria	Therapeutic agent	Type of tumor	Treatment method	Ref
*Salmonella Typhimurium*	Doxorubicin	Colorectal cancer	Chemotherapy	Ektate et al. (2018)
*Escherichia coli*	Doxorubicin	Breast cancer	Chemotherapy	Sun et al. (2021)
*Escherichia coli* Nissle1917	Doxorubicin	Breast cancer	Chemotherapy	Xie et al. (2017)
*Escherichia coli* Nissle1917	DOX and TOS	Breast cancer	Chemotherapy	Xie et al. (2018)
*Magnetotactic bacteria*MC-1	SN-38	Colorectal cancer	Chemotherapy	Felfoul et al. (2016)
*Listeria*	32P	Pancreatic cancer	Radiotherapy	Chandra et al. (2017)
*Listeria*	*TT856-1313*	Pancreatic cancer	Immunotherapy	Selvanesan et al. (2022)
*Escherichia coli*	CD47 nanobodies	Lymphoma	Immunotherapy	Chowdhury et al. (2019)
*Salmonella typhimurium*	FlaB	Colorectal cancer	Immunotherapy	Zheng et al. (2017)
*Salmonella typhimurium*	NY-ESO-1	Melanoma	Immunotherapy	Nishikawa et al. (2006)
*Salmonella typhimurium*	Cytolysin A	Colorectal cancer	Protein-based biotherapy	Jiang et al. (2013)
*Salmonella typhimurium* YB1	Indocyanine green	bladder cancer	Photothermal-therapy	Chen et al. (2019b)

### 3.2 Bacterial skeleton

Bacterial skeleton includes bacterial ghosts (BGs), and cell wall skeleton (CWs). BGs are cavities formed by lysing Gram-negative bacteria using φX174 phage lysin gene E. BGs have large inner space and intact outer walls, and can be applied as a drug delivery carrier to load cargoes of drugs, antigens, and nucleic acids (Rabea et al., 2020; Xie et al., 2020; Tabrizi et al., 2004). It has been shown that encapsulation of doxorubicin (DOX) in BG derived from *Mannheimia haemolytica* not only targets human colorectal adenocarcinoma cells (Caco-2) releasing large quantities of DOX within tumor cells but also enhances cytotoxicity against tumor cells (Paukner et al., 2004). In addition, BG retains various antigenic components of the bacteria, allowing them to also be used as a biologic agent and immune adjuvant for immunotherapy (Kudela et al., 2005). For example, when BG carries a plasmid, it is recognized and phagocytosed by antigen-presenting cells and efficiently activates CD4^+^ and CD8^+^ T cells to generate an anti-tumor immune response (Warrier et al., 2019).

CWS is prepared from bacteria by hydrothermal treatment and is an agonist of TLR2 and TLR4 and can be used as an immune adjuvant. CWS from *M. bovis* BCG has Immune activation ability (Ni et al., 2017). Researchers have found that the shape of CWS affects DC internalization (Yoo et al., 2002). The results indicated that the CWS of *Lactobacillus* rod-shaped exhibited the greatest amount of DC internalization compared to other shapes. Also, rod-shaped *Lactobacillus* CWS upregulated immunoreactive cytokines, which can be used for TME regulation or loading stimulatory signals in next-generation delivery systems (Seya et al., 2001).

### 3.3 Bacterial components

**FIGURE 3:**
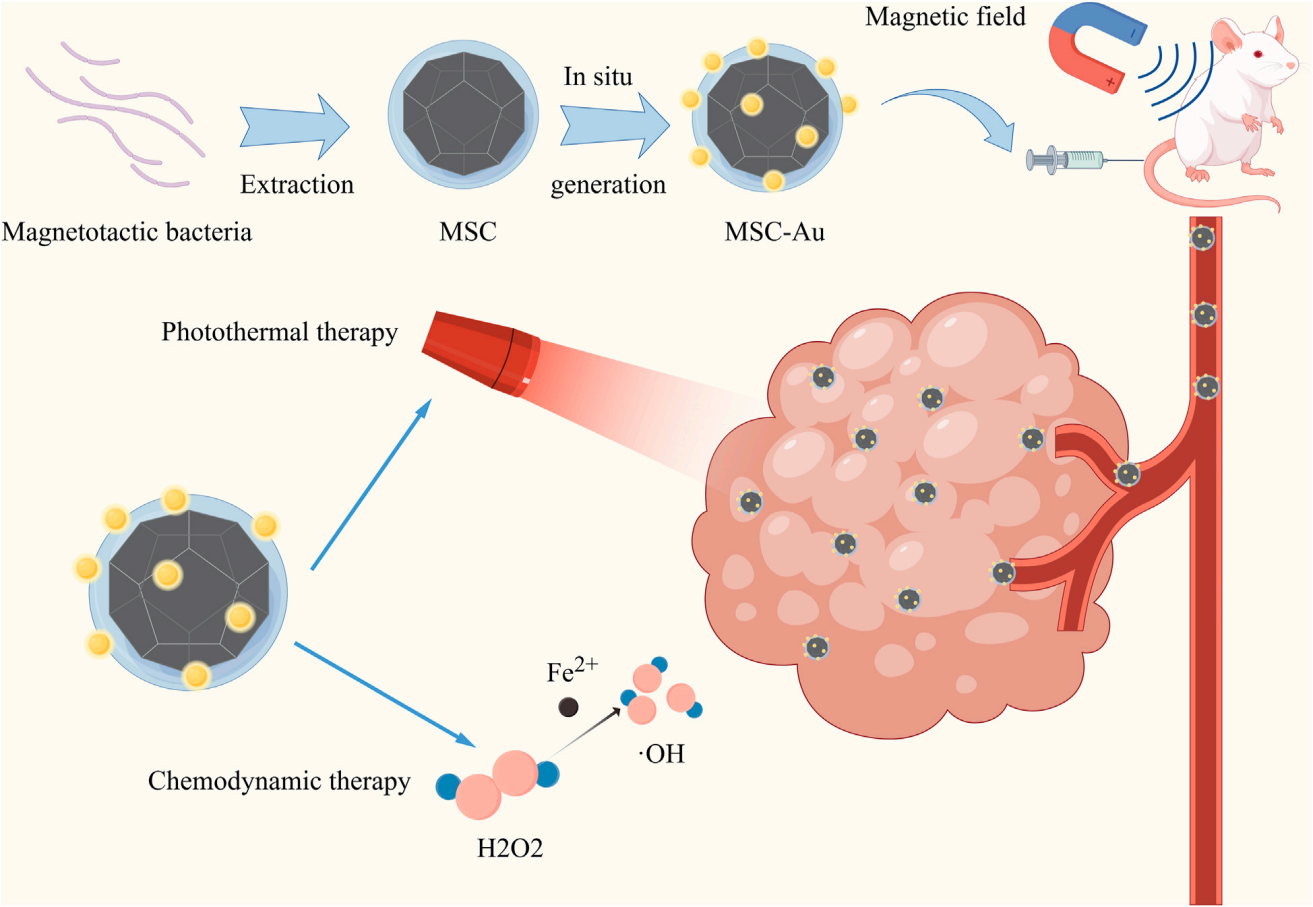
Graph illustration of the MSC was extracted from the *Magnetospirillum magneticum* AMB-1 bacterium. Subsequently, AuNPs could be grown *in situ* on this MSC. The resulting hybrid (MSC-Au) was able to target tumors in the presence of a tumor-focused magnetic field, enabling multimodal combination therapy. Reproduced with permission from (Ye et al., 2022) Reference, copyright Wiley, 2022.

Bacterial components include magnetosomes, spore microcells, and bacterial outer membrane vesicles. Magnetotactic bacteria (MTB) were found by American scholars in 1975 (Makela et al., 2022). This group of bacteria can be driven by flagella to move in the direction of magnetic lines of force and are well targeted in cancer therapy. Magnetosomes are intracellular organelles of MTB containing magnetic minerals such as magnetite Fe3O4 or greigite (Fe3S4) encapsulated by proteolipid membranes (Grünberg et al., 2004; Schüler, 2008). Magnetosome membranes are abundant in primary amine groups, which can bind certain chemotherapeutic drugs to the amine groups on the surface of the magnetosome for cancer therapy. Polymers wrapped with small molecule drugs can be attached to the magnetic vesicles to improve their drug-carrying efficiency and stability. Alternatively, chemical drugs with amine groups can be directly coupled to magnetosomes by crosslinking agents (Sun et al., 2011). Some researchers used polyethyleneimine (PEI) as a cross-linking agent to enable the magnetosomes to bind the antitumor drug DOX (loading rate of 57.7%) and small interfering RNAs (siRNAs) via a hydrazone bond (Long et al., 2018). In addition, genipin and polyglutamic acid can be selected as double cross-linking agents to load the anticancer drugs acitretin (Ara-c) and doxycycline (DNR) on the surface of the magnetic vesicles (68.4% encapsulation and 32.4% drug loading rate for Ara-c and 36.1% encapsulation and 17.9% drug loading rate for DNR) (Long et al., 2016). It was found that this magnetic vesicle drug-carrying system exhibited good stability and long-term sustained drug release, and significantly reduced the nonspecific toxicity of the drug. Meanwhile, as magnetic nanoparticles with good magnetic responsiveness, magnetosomes are able to target tumors under the action of the external magnetic field. The targeting ability can be further enhanced by combining specific targeting molecules with chemical groups on the membrane of the magnetosome (Zhang et al., 2018a). Moreover, according to the properties of magnetosomes, it is also possible to combine the delivered drugs with various therapeutic regimens, including chemodynamic therapy (CDT) (Ye et al., 2022), magnetically induced thermotherapy (Liu et al., 2012), magnetically induced photothermal therapy (PTT)and magnetically induced immunotherapy (Zhang F. et al., 2019). For example, the researchers extracted magnetosomes from the *Magnetospirillum magneticum* AMB-1 and used them as a “magnetosome chassis” (MSC) to generate gold nanoparticles *in situ*. The resulting hybrid (MSC-Gold) is capable of targeting tumors in a tumor-centric magnetic field and enables combined chemodynamic and photothermal therapies (Figure 3) (Ye et al., 2022).

Spores are formed in extreme environments and have functional components that can be used in cancer therapy. *Clostridium spp.* as a spore-producing bacterium can survive in hypoxic environments, and the spores it produces can germinate in hypoxic and necrotic areas of tumors and release carrier substances to shrink tumors (Mengesha et al., 2006; Mengesha et al., 2007). Genetically modified C. *novyi*-NT spores lack lethal toxins and have anticancer activity along with improved safety. Intratumor injection of *Clostridium histolyticum* spores and intravenous injection of *Clostridium perfringens* spores lead to lysis of tumor tissues without adverse effects on normal tissue (Kubiak and Minton, 2015). In addition, bacterial spores serve as carriers for anticancer drugs, therapeutic proteins, and cytotoxic peptides.

Minicells are the products of abnormal bacterial cell division, have a nanometer size, and are controlled by mutated minCDE genes. The capability of minicells to package multiple drugs by unidirectional diffusion, independent of their physicochemical properties, makes them ideally suited for drug delivery (MacDiarmid et al., 2009). In addition, minicells carry all the ingredients of the parental cells except chromosomal DNA and therefore cannot proliferate,which provides great safety for their use as carriers of drug delivery for the treatment of tumors (Di Ventura and Sourjik, 2011). For example, in animal studies, combining DOX with tumor-targeting minicells resulted in significant regression of *in situ* breast tumors, and no serious toxic side effects were observed.

**FIGURE 4:**
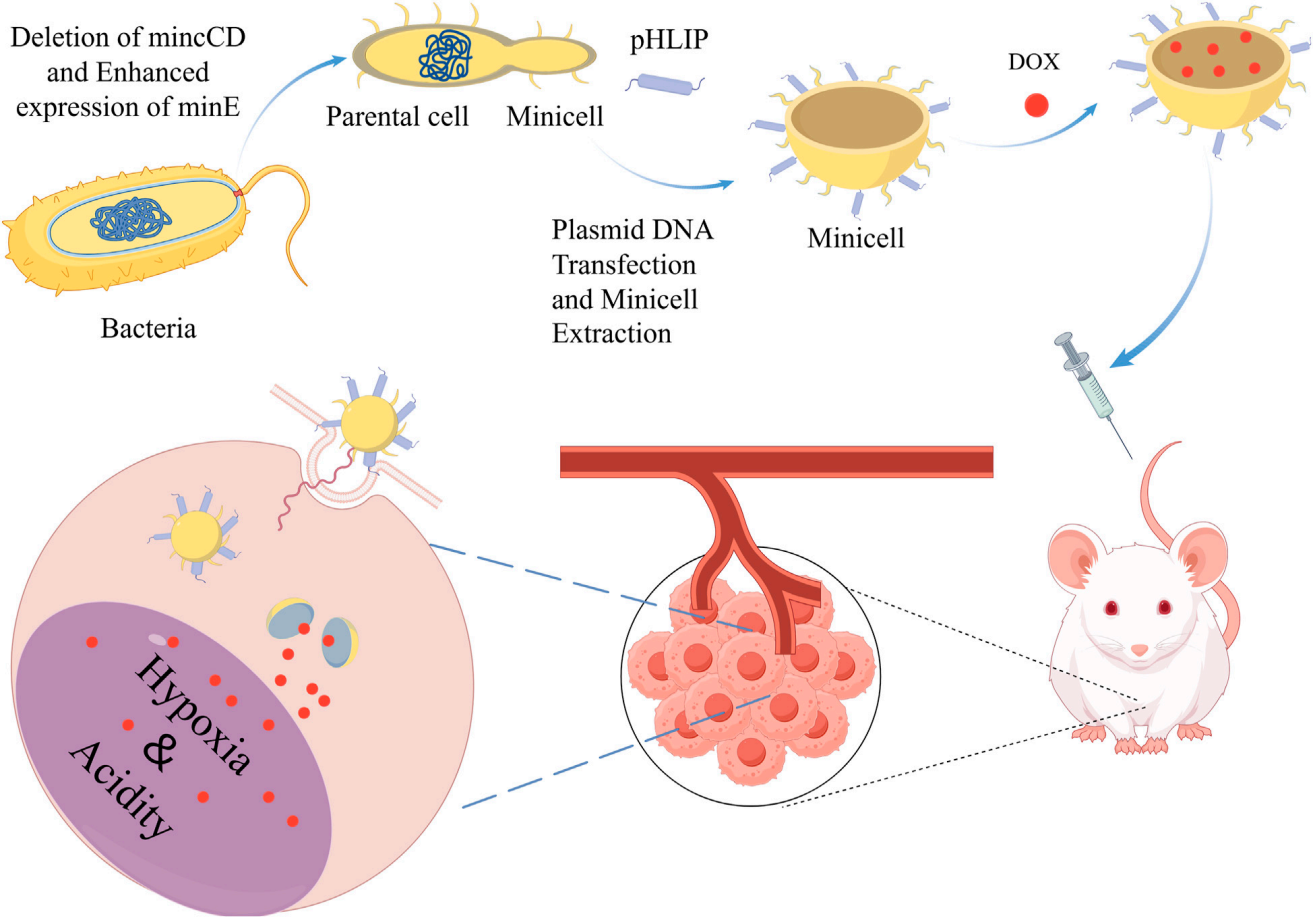
Decorative loading of pH (low) insertion peptide (pHLIP) on the surface of DOX-loaded minicells enables them to target acidic and hypoxic TME and release DOX, thereby killing tumor cells. Adapted from reference (Zhang et al., 2018b) PMC,Copyright 2018.

In addition, minicells are easy to modify, and researchers can enhance their targeting and killing of tumors by modifying minicells with ligands or antibodies. For example, MacDiarmid et al. modified minicells using antibodies that enabled the minicells to target epidermal growth factor receptors (EGFR) or human epidermal growth factor receptor 2 (HER2) receptors that are specifically overexpressed on tumor cell membranes. These modified minicells carry drugs directly into target tumor cells after packaging DOX and paclitaxel (PTX) therapeutic drugs. In addition, the researchers modified the DOX-loaded minicells so that they could target drug delivery to solid tumors (Figure 4) (Zhang et al., 2018b). Minicell-based forms of drug delivery are more effective than free drugs and can effectively reduce the toxic side effects of drugs on normal tissues (MacDiarmid et al., 2007). Currently, EGFR-modified paclitaxel-loaded *S. typhimurium* minicells have been successfully tested in phase I trials, and no patients have died during the observation period due to adverse effects (Solomon et al., 2015).

To deliver drugs, minicells have an advantage in delivering RNAi and can be efficiently loaded with siRNA and shRNA (Jivrajani and Nivsarkar, 2019). Based on this feature, MacDiarmid et al. utilized microcells to deliver siRNA/shRNA for the treatment of cancer, and the loaded siRNA/shRNA induced apoptosis in tumor cells (Jivrajani and Nivsarkar, 2016). And because their cell walls contain varying levels of LPS, they are not only expected to be a drug delivery system (Giacalone et al., 2007) but also to act as immune adjuvants for antitumor effects (Lundin and Checkoway, 2009).

Bacteria secrete membrane vesicles called bacterial extracellular vesicles (bEVs), spherical structures with a lipid bilayer containing various biomolecules from the parent bacterium. bEVs derived from the outer membrane of Gram-negative bacteria are known as outer membrane vesicles (OMVs) (Jagannadham and Chattopadhyay, 2015). The OMVs carry periplasmic and cytoplasmic components, a large number of microbe-associated molecular patterns (MAMPs) (Ellis and Kuehn, 2010), and functional components. bEVs derived from the cytoplasmic membrane of Gram-positive bacteria are known as cytoplasmic membrane vesicles (CMVs), which contain material from the cytoplasm but do not contain components that can cause an acute toxic response (e.g., LPS and other cell wall components, etc.) (Silhavy et al., 2010).

**FIGURE 5:**
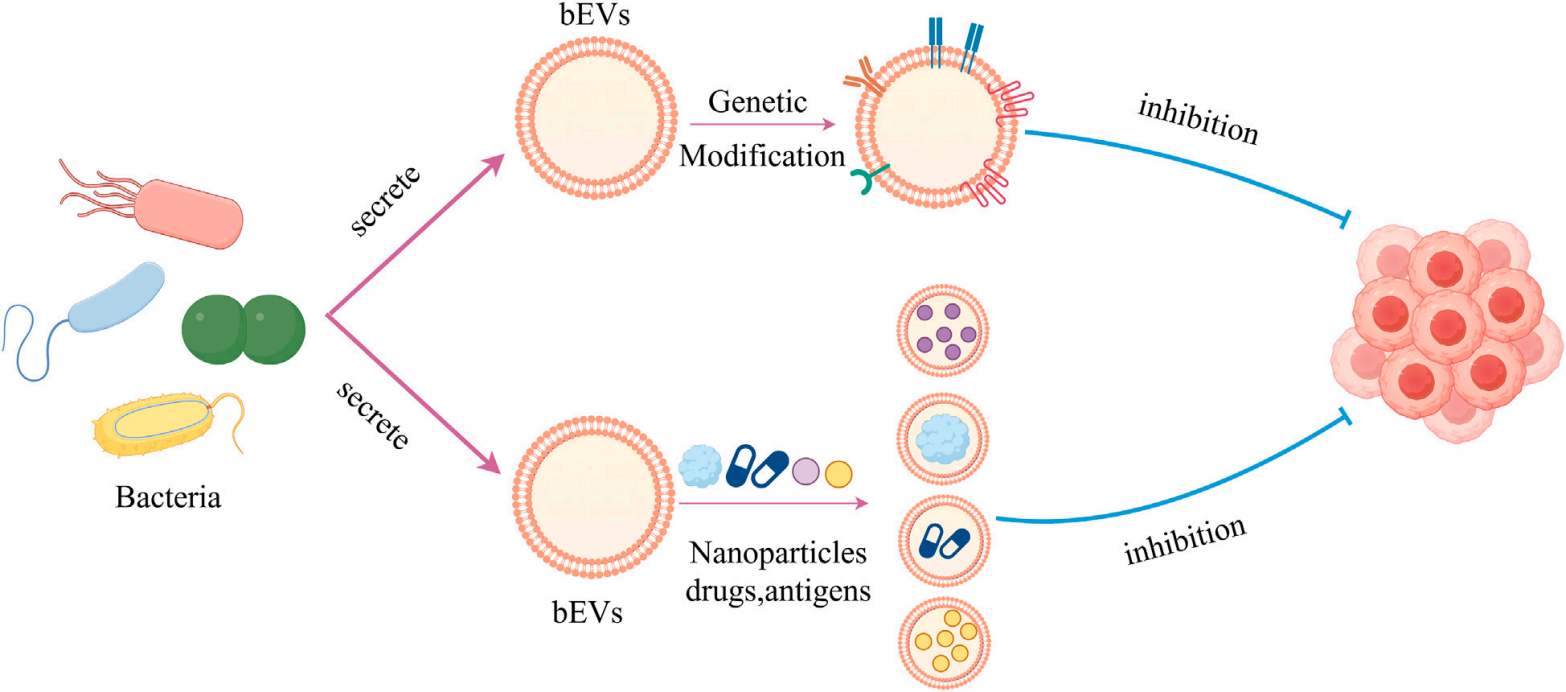
Graph showing bEVs used as anticancer drug delivery carriers as well as genetically modified for cancer therapy.

bEVs have many characteristics such as good biocompatibility and easy genetic modification, so they can be used as delivery carriers for anticancer agents. The bEVs-mediated targeted delivery system of chemotherapeutic agents is a perspective method in oncology therapy to improve effects and reduce adverse effects caused by systemic therapy. Currently, bEVs have been successfully loaded with different kinds of chemotherapeutic drugs, such as PTX, DOX, gemcitabine, and tegafur (Figure 5) (Chen Q. et al., 2019; MacDiarmid et al., 2016; Sagnella et al., 2020; Sagnella et al., 2018; Alfaleh et al., 2017).

In addition, bEVs can carry antitumor substances such as antigens, TLR agonists, and photosensitizers (Qing et al., 2020; Chen Q. et al., 2020; Wang et al., 2020). Since bEVs inherit various immunostimulatory molecules from parental bacteria, they can generate immune responses against pathogens, which gives them the potential for immunotherapy of cancer (Nagakubo et al., 2020).

Combining the capacity of bEVs to deliver drugs and immune adjuvants could further enhance their anti-tumor therapeutic potential. This holds promise for complete tumor eradication and prevention of tumor recurrence and metastasis. Examples include loading bEVs with chemotherapeutic agents (Kuerban et al., 2020) or adding photothermal agents to bEVs. Chemotherapeutic agents or PTT can lead to immune cell death in tumor cells, which, in combination with the immune properties of bEVs, can enhance the efficacy of anti-tumor therapy (Wang et al., 2020).

In conclusion, using bacterial components as delivery carriers for anticancer therapy is an innovative and evolving study (Table 2).

**TABLE 2:** Selected examples of bacterial components as delivery carriers for anticancer therapy.

Bacterial component	Therapeutic mediator	Tumor type	Therapy outcome	Ref
MTB	Dox and siRNA	Cervical cancer	Chemo-immunotherapy	Long et al. (2018)
MTB	daunorubicin	Leukemia	Chemotherapy	Long et al. (2016)
Minicell	DOX	Breast cancer	Chemotherapy	Zhang et al. (2018b)
Minicell	shRNA	Lung cancer	Immunotherapy	Jivrajani and Nivsarkar (2016)
Minicell	Paclitaxel	Breast, bladder pancreatic, prostate and lung tumors	Chemotherapy	Solomon et al. (2015)
bEV	Tegafur	Melanoma	Chemo-immunotherapy	Kuerban et al. (2020)
bEV	DOX	Neuroblastoma	Chemotherapy	Sagnella et al. (2018)
bEv	DOX	*non-small-cell lung cance*	Chemotherapy	Kuerban et al. (2020)
bEV	ICG	Melanoma	Photothermal therapy	Gu et al. (2020)
bEV	Melanin	Breast cancer	Photothermal therapy	Gujrati et al. (2019)

## 4 Bacteria combined with nanomaterials to treat cancer

Currently, drug delivery systems mediated by bacteria and their derivatives are still in the preclinical stage in the field of cancer therapy (Kavan et al., 2023; Tsujikawa et al., 2020; Nelson et al., 2023; Gerstner et al., 2022; Brahmer et al., 2021; Ramalingam et al., 2020). Research has shown that combining bacterial therapy with other treatment options can improve the efficacy and specificity of cancer treatments.

Nowadays, nanomaterials show great potential in cancer treatment and make cancer treatments more diversified. Nanomaterials have many advantages, first of all, the size of nanomaterials can be controlled (Liu et al., 2019), and can be passively or actively targeted to tumors (Zhu et al., 2022). Secondly, the large surface area/volume ratio allows it to be utilized as a drug carrier to deliver drugs (Lv et al., 2021), genes (Mei et al., 2019), and other therapeutic molecules (Wang Y. et al., 2021), and protect them from enzymatic degradation in complex physiological microenvironments. Third, since nanomaterials have a variety of functional groups on their surfaces, it is easy to modify them to enhance their properties (Hu et al., 2021). Fourth, functionalized nanomaterials can control drug release *in vivo* through various internal and external stimuli and can induce conductive photothermal or photodynamic processes and act as immunomodulators (Shen et al., 2022). Although nanoparticles have many strengths in anti-tumor therapy, they also have some weaknesses. Bacteria, on the other hand, can compensate for the shortcomings of nanotechnology and generate multiple synergistic therapeutic modalities with it. The combination of bacteriotherapy and nanomedicine technologies can be classified as follows 1) Bacteria–nanoparticle biohybrid systems, 2) Intracellular Bacteria Nanoengineering, and 3) Nanoparticle-Based Bionic Bacteria.

### 4.1 Bacteria-nanoparticle biohybrid systems

Bacteria-nanoparticle biohybrid systems have good biocompatibility degradability, and high drug loading. In this part, we will conclude the use of bacterial-nanoparticle biohybrid systems in combination with different therapeutic regimens (Table 3).

**TABLE 3:** Application of *Bacteria-nanoparticle biohybrid systems* of anticancer drugs in cancer treatment.

Bacteria	Nanomaterial	Adaptation strategy	Treatment modal	Ref
*Salmonella Typhimurium* VNP20009	Polydopamine	Coating	Photothermal-therapy	Chen et al. (2018)
*Salmonella Typhimurium* Ty21a	Gold NPs	Encapsulation	Photothermal-therapy	Kefayat et al. (2019)
EcN	PD-L1 and CTLA-4 Nanobodies	Genetic modification	Immunotherapy	Gurbatri et al. (2020)
*Salmonella Typhimurium*	Paclitaxel-loaded Liposomes	Biotin-streptavidin	Chemotherapy	Han et al. (2016)
*Salmonella Typhimurium* YS1646	Doxorubicin loaded-low-temperature sensitive liposome	Biotin-streptavidin	Chemo-immunotherap	Ektate et al. (2018)
E.coli MG1655	Doxorubicin and SPIONs loaded soft red blood Cells	Biotin-avidin-biotin	Chemotherapy	Alapan et al. (2018)
E.coli MG1655	Doxorubicin and Fe3O4 nanoparticles	Surface charge and noncovalent interactions	Chemotherapy	Park et al. (2017)
E.coli MG1655	Bi2S3 NPs	chemically modified	Radiotherapy	Pan et al. (2022)
E.coli	AIEgen	Electrostatic adsorption	PDT	Zhu et al. (2021)
*E. coli* BL21	nanoscale acoustic-sensitized particles PCN	electrostatic interaction	SDT	Wang et al. (2024)
*E. coli*	Au@Pt nanozyme	Redox reaction	Chemodynamic therapy	Zhang et al. (2021)

#### 4.1.1 Chemotherapy

Chemotherapy has been widely used in the past decades to treat various types and stages of tumors (Ouyang et al., 2020). However, standard chemotherapeutic agents have limited therapeutic efficacy owing to their lack of ability to target tumors. Besides, insufficient targeting can produce serious side effects and may lead to long-term respiratory, urinary, circulatory, neurological, and reproductive adverse effects (Zhang et al., 2022; Bhoyrul et al., 2021; Su et al., 2023). Bacteria-nanoparticle biohybrid systems with good targeting can greatly minimize systemic toxicity and improve the efficacy of chemotherapy. *Salmonella typhimurium* is one of the most researched anticancer bacterial populations, and some researchers have used biotin-streptavidin chemistry to combine *S. typhimurium* with liposomes containing paclitaxel. The study demonstrated that binding bacteria can exhibit better anti-tumor capabilities compared to the liposome-encapsulated drug alone (Han et al., 2016). Another of the most researched anticancer strains is *E. coli* Nissle 1917 (EcN), a probiotic with a high safety profile. For example, Xie et al. bound adriamycin to EcN via an acid-unstable cis-conjugated anhydride linker for tumor targeting and drug-responsive release (Xie et al., 2017).

**FIGURE 6:**
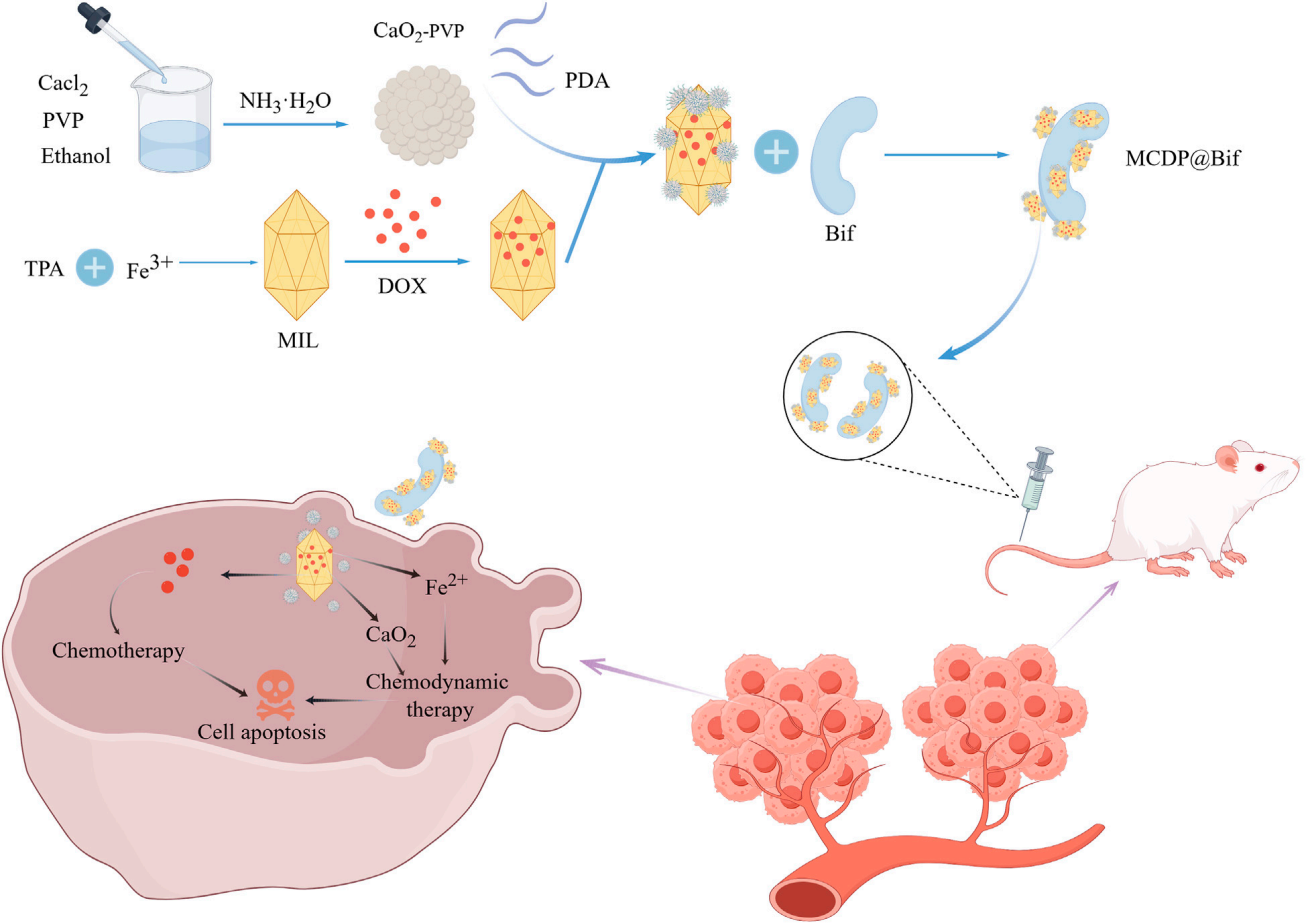
The graph depicts the CaO2 NPs and DOX were loaded onto iron-based MOFs (named as MIL), and the resulting MIL + CaO2 + DOX + polydopamine (PDA) (MCDP) NPs were attached to the surface of *Bifidobacterium infantis* (Bif) through PDA coating. Achieved CaO2 and iron ion induced chemodynamic therapy combined with DOX chemotherapeutic therapy. Adapted from reference (Li et al., 2023). Wiley, Copyright 2023.

However, the synergy between biological therapy and chemotherapy goes far beyond this. Thermobots synthesized by attaching adriamycin-containing cryo-sensitive liposomes to the surface of attenuated *Salmonella* can be used for immunotherapy of colon cancer (Ektate et al., 2018). After reaching the tumor site, the thermal robot uses high-intensity focused ultrasound at 40–42° to release adriamycin from the liposomes and stimulate polarized macrophages to change to the M1 phenotype. Additionally, the delivery of CaO2 NPs and doxorubicin (DOX) to tumor tissue using *Bifidobacterium infantis* (Bif) for synergistic chemotherapy and CDT (Figure 6) (Li et al., 2023). Bacteria can be modified not only by binding to drug-carrying nanomaterials but also by combining with natural materials to enhance their intrinsic properties. For instance, For example, Alapan et al. attached soft erythrocytes loaded with DOX and superparamagnetic iron oxide nanoparticles (SPION) to the surface of engineered *E. coli* to form microswimmers that could enable on-demand delivery and release of chemotherapeutic drugs (Alapan et al., 2018).

#### 4.1.2 Radiotherapy

**FIGURE 7:**
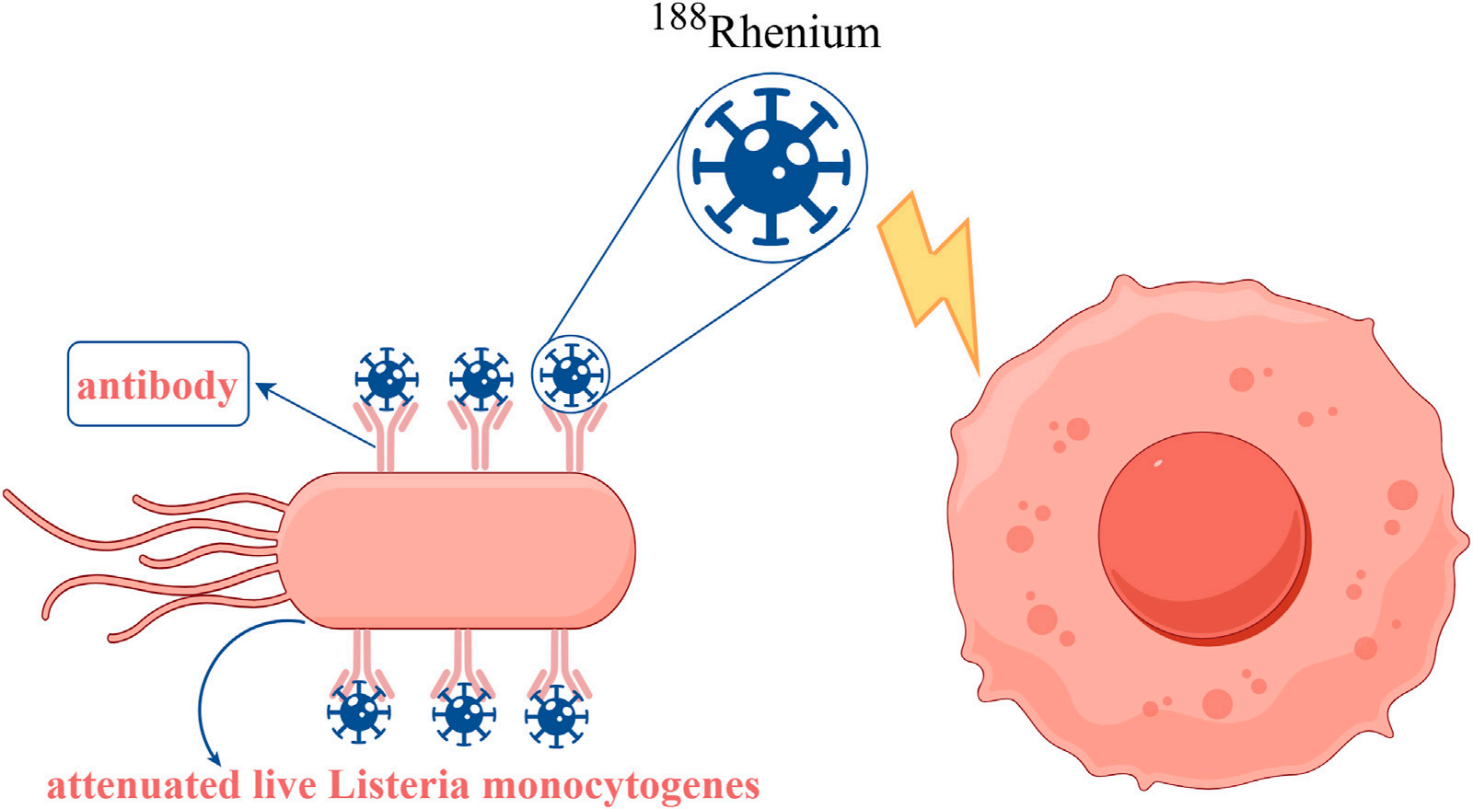
Decoration of the attenuated live *Listeria monocytogenes* conjugated with antibodies and then combined with the radioisotope 188Rhenium to produce radioactive *Listeria monocytogenes* (RL), which is effective in killing tumor cells. Adapted from reference (Quispe-Tintaya et al., 2013).

Radiotherapy is a therapeutic method that uses high-energy rays to locally irradiate tumor cells, which has a history of more than a hundred years and is an important and effective means of antitumor therapy (Kasat et al., 2010). Radiotherapy using ionizing radiation can induce the production of reactive oxygen species (ROS) causing DNA double-strand breaks in tumor cells, thereby killing the tumor (Ding et al., 2022). The oxygen content of tissues in this process plays a decisive role in the treatment (Alapan et al., 2018). Many experimental studies have shown that hypoxic cells are more resistant to ionizing radiation than normoxic cells, and therefore hypoxic and necrotic areas of tumors often lead to poor therapeutic outcomes or even treatment failure (Barker et al., 2015). Bacteria can deliver radiotherapeutic agents. For example, loading the radioisotope 188-rhenium (Figure 7) and the radiotherapeutic drug 32-phosphorus with attenuated live *Listeria monocytogenes* induces ROS production and kills tumor cells (Quispe-Tintaya et al., 2013; Chandra et al., 2017).

Cytolysin A (ClyA) overexpressed *E. coli* MG1655 was combined with bismuth trisulfide nanoparticles (BNPs). It has excellent tumor-targeting and penetration properties, which can rapidly accumulate in the lesion and achieve a deep penetration effect. It can suppress tumor proliferation through the secretion of ClyA, while the BNPs act as nano-radiological sensitizers capable of triggering a local ROS burst and DNA damage in tumor cells (Pan et al., 2022).

#### 4.1.3 Photothermal therapy

PTT is a novel tumor treatment strategy that converts light energy into heat to kill cancer cells (Xiong et al., 2023; Li et al., 2022). A variety of nanomaterials with photothermal conversion capabilities have been developed, including metallic nanomaterials, carbon-based nanomaterials, and polymer-based nanomaterials (Liu S. et al., 2020).

However, existing PTT monotherapies are not effective (Wang L. et al., 2021). To improve the effectiveness of conventional PTT Luo et al. utilized the anaerobic bacteria *Bifidobacterium shortum* and *Clostridium difficile* in combination with gold nanorods (AuNRs) to enhance the effect of PTT through the synergistic effect between the high photothermal conversion efficiency of the gold nanorods and the anaerobic bacteria’s tumor hypoxia-targeting ability (Luo et al., 2016). Additionally, PTT can cause immunogenic death of tumor cells and enhance anti-tumor immunotherapy (Shang et al., 2020). In this way, Ag2S QDs were coupled to the surface of *B. bifidum* (Zhao et al., 2022). Engineered bacteria can promote the secretion of immune factors and activate T-cells for immunotherapy. Ag2S QDs have a high efficiency of photothermal conversion, which can induce the production of tumor-specific antigens by ICDs, and further enhance the effect of immunotherapy.

#### 4.1.4 Immunotherapy

Tumor immunotherapy is an innovative treatment method that uses the body’s own immune system to fight cancer. With the continuous advancement of medicine, it has become the fourth most popular cancer treatment after surgery, chemotherapy, and radiotherapy. The discovery by researchers in the 19th century that *coli* toxins can trigger the activity of the immune system against tumors set the precedent for bacterial immunotherapy (Karbach et al., 2012).

In recent years, significant progress has been made in bacterial immunotherapy, and some of these therapies have successfully entered clinical development. For example, attenuated bacteria are programmed to express and deliver various tumor suppressor cytokines to enhance anti-tumor immunity and induce apoptosis in tumor cells (Duong et al., 2019). Other immunotherapeutic agents, such as cytotoxic proteins (He et al., 2019), antigens, and antibodies (Nishikawa et al., 2006), have also been overexpressed in attenuated engineered bacteria and used for tumor therapy.

Bacteria have immunomodulatory effects and have great potential in cancer immunotherapy. Wang et al. further enhanced the immunotherapeutic effect of *Salmonella* by combining cationic polymer nanoparticles coated on its surface. The complexes could adsorb tumor antigens and transfer them to the periphery of the tumor. The crosstalk between antigens and dendritic cells increased significantly when the antigens were transferred to the periphery of the tumor. Chemotherapy, radiotherapy, and photothermal effects can enhance cancer immunotherapy by inducing immunogenic death and releasing cancer antigens (Huang et al., 2021; Fu et al., 2022; Liu et al., 2023), and the combination of bacterial immunotherapy and the aforementioned therapeutic regimens has become a hot topic of current research.

#### 4.1.5 Other

**FIGURE 8:**
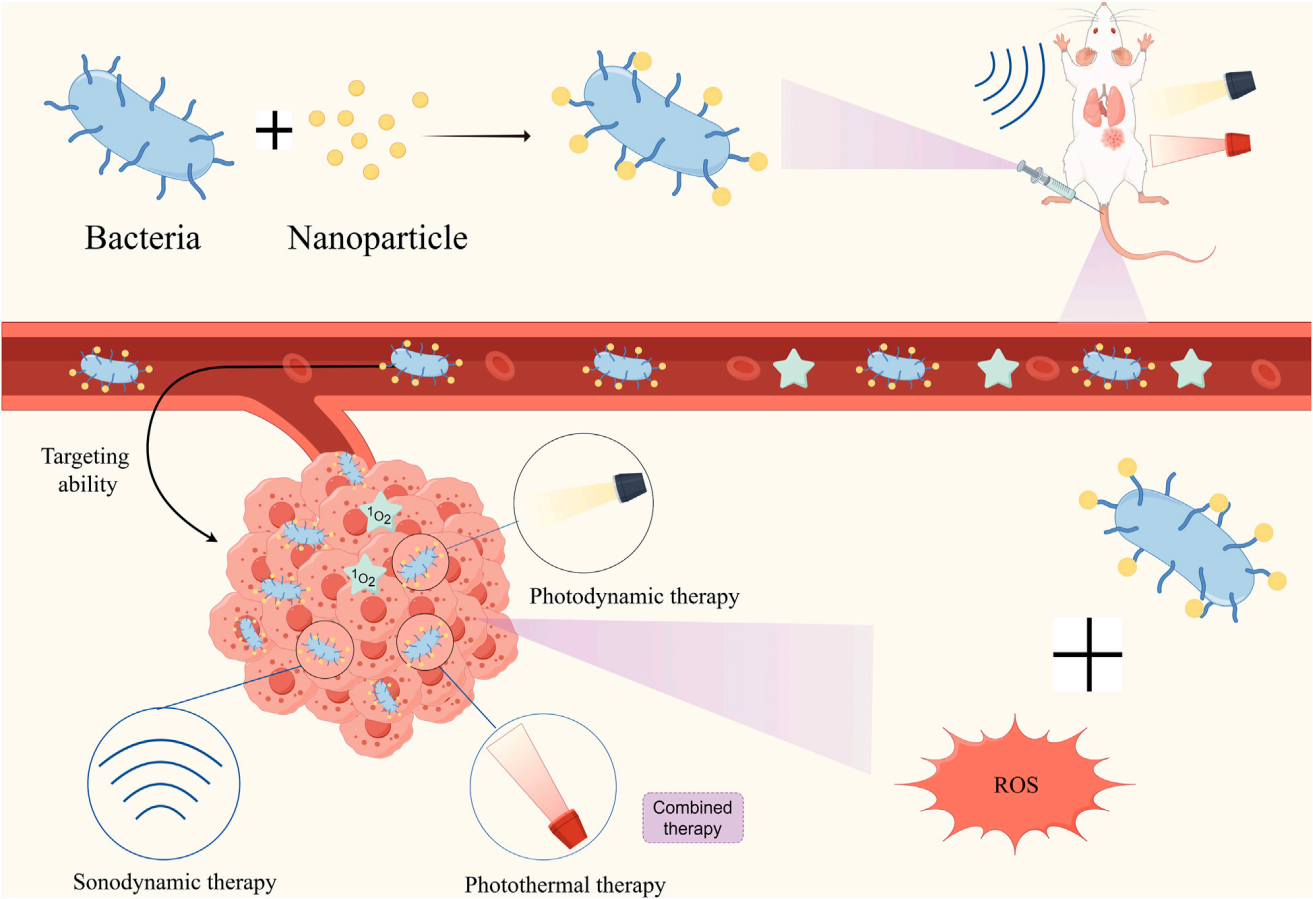
Diagrammatic depiction of combining bacteria with nanomaterials to make ROS-based cancer therapies (PDT CDT SDT) more effective using bacterial targeting properties.

Although tumor cells can tolerate higher concentrations of ROS, it can also be devastating to cancer cells when the amount of ROS exceeds the cellular tolerance threshold (Yang B. et al., 2020). Based on these mechanisms, a range of ROS-based therapies have been developed, such as radiation therapy, Photodynamic therapy (PDT), sonodynamic therapy (SDT), and CDT. Combining the above treatment options with bacterial therapies may also produce even better cancer outcomes (Figure 8).

PDT is a treatment that utilizes photosensitizers and specific light sources to selectively kill cancer cells (Karges et al., 2019; Karges, 2022). The photosensitizer or material absorbs light energy and converts it into chemical energy, generating reactive oxygen species capable of killing tumor cells without affecting normal cells (Liu et al., 2015). The use of bacteria to construct an oxygen self-supply system can strengthen the therapeutic efficacy of PDT. For example, by coupling *Chlorella* with chlorine-6 nanoparticles (Wang H. et al., 2022), *Chlorella* can produce O2 under 660 nm light irradiation to enhance PDT, and then the enhanced PDT can lyse *Chlorella* to release adjuvants on the bacterial surface and activate biotherapeutic combined immunotherapy. Type I photosensitizers can produce ROS in the absence of O2, which solves the problem of photosensitization that occurs with conventional type II photosensitizers in oxygen-depleted tumors. This solves the problem of decreased efficiency of photosensitization in oxygen-depleted tumors with conventional type II photosensitizers. Combining type I PDTs with bacteria is of great therapeutic importance for the treatment of tumors (Zhu et al., 2021). Zhu et al. developed a novel bacterial-based AIEgen (TBP-2) nanohybrid system (AE), and the AIEgen nano bio-hybrids significantly inhibited the growth of *in situ* colon tumors by facilitating the generation of ROS in the hypoxic tumor region under light irradiation.

SDT is an up-and-coming non-invasive tumor treatment that effectively kills tumor cells by activating acoustic sensitizers through ultrasound to produce ROS at the tumor site (Li et al., 2021; Gong and Dai, 2021; Son et al., 2020). It is an effective cancer treatment because of its good tissue penetration, non-invasiveness, and low toxicity (Li et al., 2020). The therapeutic efficacy of SDT is closely dependent on the concentration of oxygen in the tumor, as most acoustic sensitizers require oxygen as a feedstock to generate ROS such as single-linear oxygen under ultrasonic excitation. Plasmid transfection of *E. coli* BL21 was performed to overexpress catalase, followed by nanoscale acoustic-sensitized particles PCN synthesized from acoustic-sensitizing molecules tetrakis (4-carboxyphenyl) porphyrin (TCPP) and zirconium clusters (Zr6) NPs was adsorbed onto the bacterial surface by electrostatic interaction to obtain the multifunctional biohybrid *E.coli*-pE@PCN. After intravenous injection, *E.coli*-pE@PCN showed good tumor targeting and penetration ability, which not only could continuously express catalase to alleviate the tumor hypoxia but also promoted the enrichment and expanded distribution of carried acoustic sensitizers in the tumor site, thus triggering an effective SDT (Wang et al., 2024).

CDT is a promising therapeutic modality that utilizes endogenous overexpression of H2O2 in tumors to generate toxic hydroxyl radicals (·OH) via the Fenton/Fenton-like reaction (Wang Z. et al., 2022; Zhu et al., 2023). Due to the high H2O2 properties of TME, CDT therapy can autonomously generate ROS in TME. Nevertheless, the efficacy of CDT is often restricted by tumor antioxidant capacity (Zhou et al., 2021). By modifying Au@Pt nanozymes (Bac-Au@Pt) on the bacterial surface, the nano-system can effectively release ROS into tumor cells due to the targeting ability of the bacteria and the catalytic properties of Au@Pt nanozymes under the acidic environment. In addition, the complex reduces the antioxidative capacity of the tumor thus further increasing the CDT effect (Zhang et al., 2021).

### 4.2 Intracellular bacteria nanoengineering

Nanoparticles can be obtained by synthesizing them by various chemical methods, but chemical synthesis methods have disadvantages such as high toxicity, low purity, and poor biocompatibility. Synthesizing various nanoparticles by using bacteria as factories or base materials can solve the above problems, and thus has attracted attention. For example, metal nanoparticles can be synthesized naturally in bacteria or through genetic engineering, and most of the bacteria-mediated synthesis of metal nanoparticles has been shown to have antitumor activity *in vitro* (Kang et al., 2008; Park et al., 2010).

Almalki and Khalifa, (2020) synthesized silver nanoparticles (AgNPs) with diameters between 15 and 40 nm from *Bacillus sphaericus* KFU36. They found that the synthesized AgNPs could enter the TME and aggregate to induce significant tumor cell apoptosis. AgNPs synthesized by Yang et al. promoted intracellular ROS production in tumor cells, which increased lipid peroxidation and led to significant tumor shrinkage (Yang et al., 2020b). Vairavel uses *Enterococcus faecalis* to synthesize gold nanoparticles intracellularly, which inhibits tumor cell proliferation by generating ROS in tumor cells (Vairavel et al., 2020). Rajkumar et al. synthesized selenium nanoparticles (SeNPs) using *Pseudomonas Schizosaccharomyces*, which significantly inhibited tumor angiogenesis and killed tumor cells at low concentrations (Rajkumar et al., 2020).

### 4.3 Nanoparticle-based bionic bacteria

**FIGURE 9:**
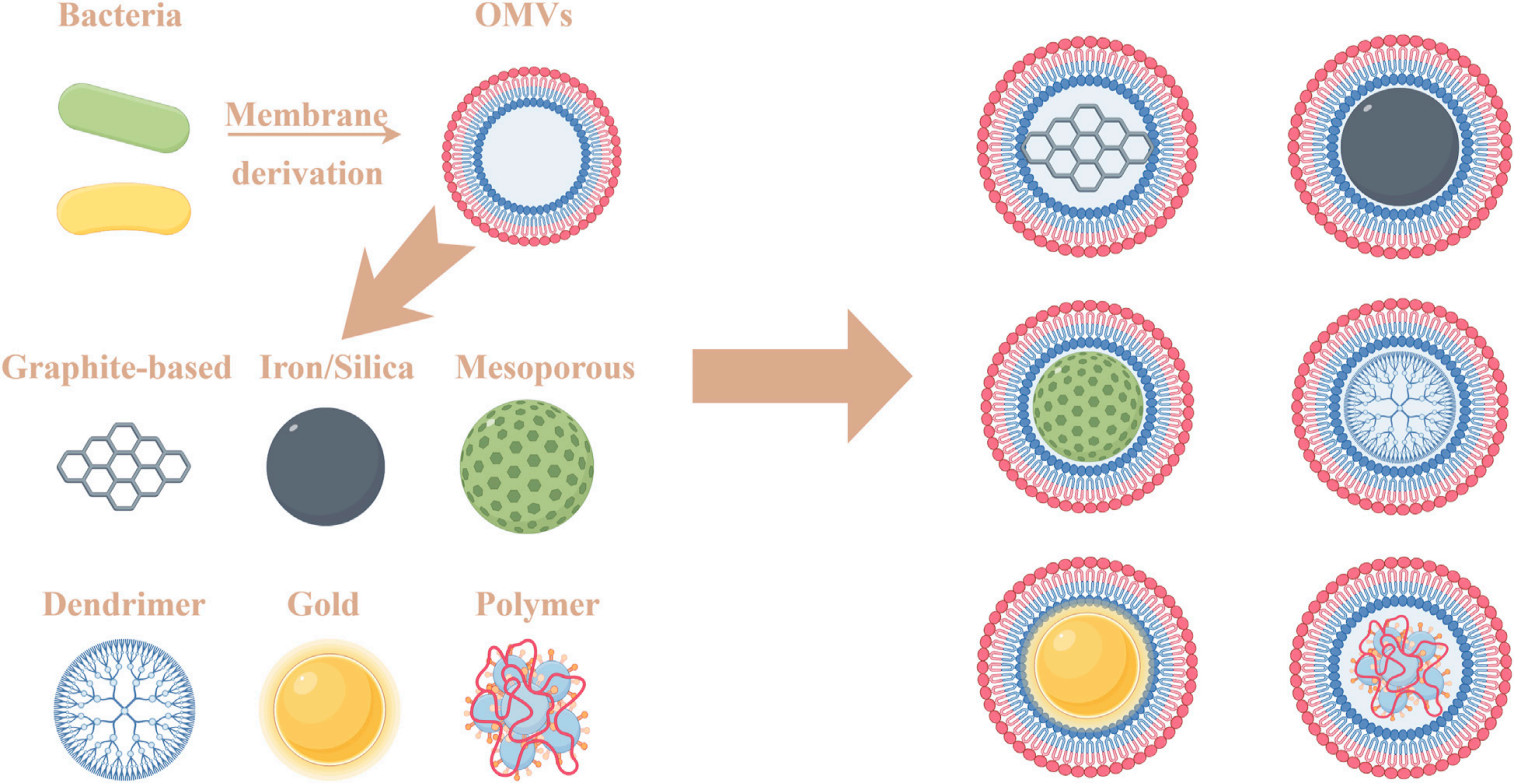
Schematic representation of OMVs coatings of different nanomaterials.

Biomimetic nanoparticles (NPs) formed using cell membranes encapsulating NPs are a promising biomedical material. Among various types of cell membranes, OMV shows great potential in the biomedical field (Qing et al., 2019). Coating NPs with bacterial OMVs retains the intrinsic properties of synthetic NPs and also enhances the properties possessed by the OMVs (Figure 9). For example, fusion of drug-carrying liposomes with OMVs can further enhance the drug-carrying capacity (Piffoux et al., 2018). Chen Q. et al. (2019) used bacterial OMVs to encapsulate drug-loaded polymeric micelles. The OMVs activated the host immune response and combined with the chemotherapeutic drugs contained in the polymeric micelles, effectively inhibiting tumor growth. In a report by Wang et al. (2020), OMVs and B16-F10 cancer cell membranes were successfully coated on hollow polydopamine (HPDA) NPs. This material could combine immunotherapy and PTT to treat melanoma. Researchers genetically modified a bacterial strain by expressing a tyrosinase transgene and generated bacterial microvesicles loaded with the biopolymer melanin. These bioengineered bacterial microvesicles were used as bio-nano-heaters for not only *in vitro* and *in vivo* tumor imaging but also tumor growth inhibition under near-infrared light irradiation (Gujrati et al., 2019).

Chen et al. formed a hybrid cell membrane by fusing a tumor cell membrane with a bacterial cytoplasmic membrane. By encapsulating polylactic acid-glycolic acid copolymer (PLGA) nanoparticles within the hybridized membrane, antigens and adjuvants from both cell membrane sources could be delivered simultaneously, thereby enhancing the innate immune response. Improved anti-tumor effect while avoiding side effects (Chen et al., 2021). In addition, biofilm components extracted from bacteria can also be used as nanoparticles for drug delivery. Yi et al. assembled rhamnolipids extracted from *Pseudomonas aeruginosa* with the photosensitizer magnesium chlorophyllate (Pba), and the assembled nanomaterials showed a significant increase in accumulation in tumor tissues and demonstrated significant tumor inhibition (Yi et al., 2019). These encouraging studies suggest that nanoparticle-based bionic bacteria hold promise for cancer therapy.

## 5 Strategies for combining nanomaterials and bacterial carriers

The binding of nanomaterials to bacteria is a prerequisite for synergistic therapy. Here, we summarize the different ways in which bacteria and their components can be combined with nanomaterials and the resulting biological functions.

### 5.1 Bioconjugation

Biological strategies allow for the fabrication of nanohybrids via biorecognition processes (e.g., antigen/antibody and biotin/streptavidin) and metabolic pathways (Mostaghaci et al., 2017). Among them, streptavidin and biotin are the most popular binding pairs used in biotechnology applications, which are highly selective and have strong interactions to withstand extreme temperatures (Tm of 112°C), pH, denaturants, and enzymes (Dundas et al., 2013). Suh et al. used this method to attach streptavidin-labeled drug-carrying poly (lactic-ethanolic acid) nanoparticles to *Salmonella* typhimurium VNP20009, which formed a biotin surface. They demonstrated a 100-fold increase in the ability to target tumors without any external driver (Suh et al., 2019).

To attach nanoparticles to bacteria through streptavidin-biotin interactions, the utilization of genetically engineered bacteria is an alternative method of bioconjugation. For example, by increasing the binding affinity and introducing new binding sites, bacteria can be preferentially adsorbed to specific nanoparticles (Vizsnyiczai et al., 2020). By designing genetic circuits, it is possible to control the binding of bacteria to nanomaterials and maintain bacterial viability and proliferation. Bioconjugate gene modification with high loading capacity can solve the problem of mutual repulsion between negatively charged nanoparticles and negatively charged bacteria, making it a potential platform for a wide range of applications (Yang et al., 2020c).

### 5.2 Physical

Physical strategies involve the use of bacteria as carriers to load or anchor materials or drugs to the outer surface of the bacteria, including electroporation (Ding et al., 2021), electrostatic interactions (Xie et al., 2021), impregnation (Kuang et al., 2023), or membrane coating (Wu et al., 2019).

Electroporation techniques can facilitate the entry of therapeutic drugs into bacteria by applying an enhanced electric field to increase the permeability of the cell membrane. Electrostatic interactions, dip-coating processes, and cell membrane coating techniques can also be used to construct nano-biological mixtures. by anchoring nanomaterials to the outer surface of bacteria. Of these, electrostatic interactions are relatively flexible in the construction of nanohybrid materials, and using this method bacteria can be used with virtually any charged nanoparticle, polyelectrolyte, protein, or polysaccharide.

Although physical strategies have the advantages of simplicity and rapidity in constructing nanohybrid materials, they also have some drawbacks. For example, electroporation affects bacterial activity, while electrostatic interactions are less stable *in vivo* applications due to the prevailing competitive reactions (Zoaby et al., 2017).

### 5.3 Chemistry

Chemical groups on the surface of bacteria make it possible to bind bacteria and nanoparticles, which can provide a more stable bond between bacteria and nanomaterials through chemical bonding. To date, many chemical reactions have been employed to bind nanomaterials to bacterial surfaces, including carbodiimide chemistry (Taherkhani et al., 2014), Michael addition reactions (Xing et al., 2021), intermolecular disulfide bond cross-linking (Liu L. et al., 2020), host-guest chemistry (Chen QW. et al., 2020), Schiff base reactions (Chen QW. et al., 2020), and click chemistry (Xie et al., 2018). Of these, carbodiimide chemistry has received much attention due to the enrichment of bacterial surfaces with amino or carboxyl groups.

### 5.4 Biomineralization

Biomineralization, the process by which organisms utilize mineral elements, is a promising approach to materials engineering. Unlike the method of combining nanoparticles with bacterial surfaces, this approach can assist bacteria form nanoparticles directly. For example, Chen et al. developed a self-mineralizing photothermal bacterium by biomineralizing palladium nanoparticles (PdNPs) on the surface of the parthenogenetic anaerobic bacterium *Shewanella oneidensis* MR-1 (188). Biomineralization consists of two main methods: biologically induced and biologically controlled. Mineralization during biologically induced processes usually occurs on the bacterial surface and is not directly regulated by genes. In turn, biologically controlled mineralization processes, such as nucleation, growth, and localization, can be regulated by genes and occur both intracellularly and extracellularly. Magnetic nanoparticles (magnetosomes) produced by MTB are a prime example of biologically controlled mineralization (Liu et al., 2024).

### 5.5 Bionic nanoparticles encapsulated by bacterial outer membrane vesicles

Currently, there are three main approaches to preparing bacterial OMV-coated nanomaterials. The first method, physical co-extrusion involves the repeated extrusion of a mixture of bacterial outer vesicles and nanoparticles through a thin porous membrane of nanoscale porous polycarbonate using an Avanti micro-extruder (Wu et al., 2020). Although this process is very efficient for the production of OMV-NPs, the deposition of raw materials on filters causes losses and makes it impractical for wide-scale production.

The second method, the ultrasonic fusion method, in which ultrasonic energy is used to promote the encapsulation of nanomaterials by extracellular membrane vesicles, seems to have solved the production scale problem (Zhang Y. et al., 2019). However, encapsulation inhomogeneity and dimensional variations are the shortcomings of this technique.

The third method is microfluidic electroporation (Rao et al., 2017). technique that involves mixing cell membranes with nanoparticles and then facilitating the entry of the nanoparticles into the cell membranes through microfluidic electroporation. It has the advantages of high accuracy and scalability, uniform product size, reproducibility, and stability.

## 6 Challenges and future directions

Novel therapeutic agents for the treatment of cancer are emerging, and therefore, drug delivery platforms need to evolve to further improve therapeutic efficacy and address the new challenges. Current findings suggest that bacteria and their derivative-mediated drug delivery systems may become a major asset in the fight against cancer. With the natural properties of bacterial carriers, such as tumor targeting, ease of genetic modification, and activation of immune responses, researchers can develop new strategies to further optimize bacterial carriers and enhance their anti-cancer efficacy. One innovative and effective strategy is to combine nanomaterials with bacterial carriers to form a hybrid system. The nanomaterials can enhance the therapeutic effect of the bacterial carriers, while the bacterial carriers can enhance the therapeutic effect of the nanomaterials, thus maximizing the synergistic therapeutic effect. The hybrid system has been shown to perform multimodal synergistic therapies and has shown encouraging results in improving cancer treatment outcomes.

However, research on bacterial carriers is still in the preclinical stage, and much effort is still needed to address some scientific issues before the translation to the clinic can be truly accomplished. Some of the shortcomings that need to be overcome and future research directions mainly include,

(i) The mechanism of interaction between bacteria and their hosts is complex and may lead to unforeseen consequences. Research on relevant mechanisms of action should be improved, and bacterial toxicity reduction programs should be continuously improved without affecting the anti-cancer properties of bacteria.
(ii) It is difficult to quantify the dosage of bacterial therapy. If certain strains of bacteria are out of the normal range, it may disrupt the balance of the bacterial flora, leading to dysbiosis and diseases. Improvement of *in vivo* monitoring strategies to monitor the distribution of bacteria in the body as a means of assessing their safety in the phase.
(iii) Currently, a single bacterium is mostly used for cancer treatment, and synergistic treatment by forming hybrids of multiple bacteria could be considered.
(iv) Using patient-specific bacterial derivatives, personalized medical protocols can be adapted to provide more targeted and effective treatments for cancer patients.
(v) The route of administration is also a major limitation of bacterial therapies. Systemic injection of bacteria can lead to serious complications, and the acidic environment of the stomach during oral administration limits the accumulation of bacteria in the target area. Enhanced research on routes of administration could improve efficacy and reduce the likelihood of adverse effects
(vi) Establishing reliable methods for the mass production of bacteria-nanomaterials hybrids is of great importance and key to reducing the cost and improving the accessibility of therapies.

Although the main focus of this review is on the use of bacteria for cancer treatment, the use of bacteria as *in vivo* diagnostics is another promising approach with the rapid advances in nanomedicine and synthetic biology. Overall, bacteria-based therapies could provide a more powerful and targeted treatment for cancer. It is believed that with the concerted efforts of a multidisciplinary team of clinicians and scientific researchers, bacterial carrier drug delivery systems are expected to become another powerful weapon in the clinic in the near future.
